# The SlDOF3.4-SlCDF4 module improves tomato growth under low-nitrogen conditions

**DOI:** 10.1093/hr/uhag020

**Published:** 2026-01-20

**Authors:** Senlin Zeng, Juan Du, Xudong Sun, Lamei Zheng, Xu Li, Sodmergen, Kunzhi Li, Huini Xu

**Affiliations:** Faculty of Life Science and Technology, Kunming University of Science and Technology, Jingming South Street, Kunming, Yunnan 650224, China; Faculty of Life Science and Technology, Kunming University of Science and Technology, Jingming South Street, Kunming, Yunnan 650224, China; Yunnan Key Laboratory of Crop Wild Relatives Omics, Institute of Tibetan Plateau Research at Kunming, The Germplasm Bank of Wild Species, Kunming Institute of Botany, Chinese Academy of Sciences, Kunming 650201, China; Faculty of Life Science and Technology, Kunming University of Science and Technology, Jingming South Street, Kunming, Yunnan 650224, China; Faculty of Life Science and Technology, Kunming University of Science and Technology, Jingming South Street, Kunming, Yunnan 650224, China; Faculty of Life Science and Technology, Kunming University of Science and Technology, Jingming South Street, Kunming, Yunnan 650224, China; The Academy for Cell and Life Health, Kunming University of Science and Technology, Jingming South Street, Kunming, Yunnan 650224, China; Faculty of Life Science and Technology, Kunming University of Science and Technology, Jingming South Street, Kunming, Yunnan 650224, China; Faculty of Life Science and Technology, Kunming University of Science and Technology, Jingming South Street, Kunming, Yunnan 650224, China

## Abstract

DNA-binding with one finger (DOF) proteins are plant-specific transcription factors (TFs) that play critical roles in plant growth and development, including nitrogen metabolism, but the roles of these TFs in the nitrogen response of tomato (*Solanum lycopersicum*) remain largely unexplored. Here, we show that overexpressing the DOF gene *SlDOF3.4* enhanced the growth of tomato seedlings under low nitrogen (LN) conditions, resulting in longer roots and greater biomass accumulation. Multiple assays demonstrated that SlDOF3.4 interacts with another DOF family member, SlCDF4, and that both TFs bind to the promoters of the N-assimilation gene *Glutamine synthetase* (*SlGS*) and the jasmonic acid (JA) biosynthesis gene *Lipoxygenase* (*SlLOXD*), suggesting that SlDOF3.4 and SlCDF4 cooperatively regulate nitrogen assimilation and JA biosynthesis. In support of this notion, co-expressing *SlCDF4* and *SlDOF3*.*4* enhanced the binding activity of SlDOF3.4 to the *SlGS* and *SlLOXD* promoters in a dual-luciferase reporter assay. Under LN conditions, genes related to nitrogen assimilation and JA biosynthesis were markedly upregulated in *SlDOF3.4*-overexpressing and *SlCDF4-*overexpressing tomato plants. Knockout of *SlCDF4* impaired plant growth under LN conditions, a phenotype that was partially alleviated by treatment with methyl jasmonate. These results provide insight into the roles of DOF TFs in nitrogen assimilation and JA biosynthesis in crops.

## Introduction

Nitrogen (N) fertilizers are widely used to increase crop productivity. However, a significant portion of applied N is unused by plants and runs off into the soil, leading to substantial environmental pollution [1]. There is therefore an urgent need to develop crop varieties with enhanced N use efficiency (NUE) to curtail the excessive use of N fertilizers and promote sustainable agriculture. Improving NUE will require a comprehensive understanding of the mechanisms by which specific crops respond to low N (LN) conditions. Nitrate (${\mathrm{NO}}_3^{-}$), the major N source for most land plants, is taken up by nitrate transporters in the root [2] and reduced to ammonium (NH_4_^+^) by nitrate reductase (NR) and nitrite reductase (NiR). The resulting NH_4_^+^ is assimilated into glutamine and glutamic acid by glutamine synthetase (GS) and glutamate synthase (GOGAT), respectively, and subsequently used to synthesize additional amino acids. Considerable efforts have been devoted in the last years to identify transcription factors (TFs) and regulatory networks that control nitrate responses. Gene regulatory network analysis allowed for identification of the endodermis as a cell type enriched in regulatory interactions, with ABF2 and ABF3 transcription factors acting as key regulators of gene expression in the endodermis and lateral root growth in response to nitrate [3]. The transient TF–target interactions captured uncover the early mode-of-action of NIN-LIKE PROTEIN 7 (NLP7), a master regulator of the nitrogen signaling pathway in plants [4].

Plant hormones serve as essential signaling molecules in regulating plant growth, development, and stress responses. Among phytohormones, jasmonic acid (JA) is known to regulate root growth and modulate root system architecture under nutrient deficiency [5]. Low N stress increases the fresh weight of roots, lateral root density, and root surface area, as well as enhancing the accumulation of indole-3-acetic acid and JA in Strawberry (*Fragaria × ananassa Duch.*) roots [6]. Several transcriptome studies have shown that JA biosynthesis genes were differentially expressed under LN stress [6–8]. The pathway mediated by JA, biosynthesized via 13-lipoxygenases (LOX), plays a central role in both plant development and defense [9]. LOX plays a critical role in plant biotic and abiotic stress responses by mediating lipid peroxidation and the production of JA [10]. However, little is known about the role of JA in tomato (*Solanum lycopersicum*) under LN stress.

DOF (DNA-binding with one finger) domain proteins are plant-specific transcription factors with a highly conserved DNA-binding domain, which presumably includes a single C-2-C-2 zinc finger [11]. DOFs are plant-specific TFs that typically consist of an N-terminal conserved DNA binding domain and a C-terminal domain for transcriptional regulation [12]. These TFs bind to the DOF CORE element (5′-(A/T)AAAG-3′) in their target genes [13]. DOF TFs are involved in multiple aspects of plant growth and development, including N metabolism [14, 15]. *TaDof1* expression was higher under low and normal N treatments than under high N treatment in wheat (*Triticum aestivum*) [16]. OsDOF11 promotes N uptake and helps maintain a constant ratio of fresh weight to dry weight in rice (*Oryza sativa*) seedlings and a suitable effective leaf-blade area during the flowering stage [17]. Overexpressing *PnDof30* significantly improved the growth of transgenic Arabidopsis (*Arabidopsis thaliana*) plants and enhanced N metabolism under LN conditions [18]. OsDOF18 was shown to mediate ammonium transport and N distribution, thus affecting NUE in rice [19].

Cycling DOF transcription factors (CDFs) regulate different aspects of plant growth and development such as photoperiodic flowering-time control and root and shoot growth [20]. Ectopic expression of the *AtCDF1* transcription factor in potato enhances tuber starch and amino acid contents and yield under open field conditions [21]. Arabidopsis CDF3 transcription factor increases carbon and nitrogen assimilation and yield in trans-grafted tomato plants [22]. Potato StCDF1 directly represses expression of NR/NIA, which catalyzes the first reduction step in nitrate assimilation. StCDF1 knock-down lines performed better in N-limiting conditions, and this phenotype correlated with derepressed *StNR* expression [23]. Overexpression of SlCDF4 promoted changes in the profile of carbon and nitrogen compounds related to fruit quality [24]. The AtCDF3 is involved in nitrogen responses and improves NUE in tomato [25]. However, the roles of DOFs in tomato under LN have not been investigated.

There are 34 *DOF* genes in the tomato genome [26], including five members of the *Cycling DOF* (*CDF*) subfamily [27]. However, whether and how SlDOFs participate in N responses remain largely unknown. In our previous RNA-seq [28] and RT-qPCR analyses, we observed a significant increase in *SlDOF3.4* transcript levels in the leaves of tomato plants under LN conditions, prompting us to investigate the potential role of this gene in plant acclimation to LN. In the present study, we examined the potential role of *SlDOF3.4* in the LN stress tolerance mechanism of tomato. Transgenic tomato seedlings overexpressing *SlDOF3.4* (*SlDOF3.4*-OE) were more tolerant to LN conditions than wild-type (WT) seedlings. Through multiple assays, we determined that SlDOF3.4 interacts with SlCDF4 and that both TFs bind to the promoters of the N-assimilation gene *Glutamine synthetase* (*SlGS*) and the JA biosynthesis gene *Lipoxygenase* (*SlLOXD*). Therefore, the SlDOF3.4-SlCDF4 complex enhances LN tolerance in tomato seedlings.

## Results

### *SlDOF3.4* improves the growth of tomato seedlings under LN stress

RT-qPCR analysis revealed a significant increase in *SlDOF3.4* transcript levels in the leaves of tomato plants under LN conditions (Fig. S1), prompting us to investigate the potential role of this gene in plant responses to LN. Phylogenetic analysis indicated that SlDOF3.4 shared the highest sequence identity (67.68%) with StDOF3.4 from potato (*Solanum tuberosum*) and was classified into subgroup V (Fig. S2A). The highly conserved zf-Dof domain, a characteristic feature of the DOF family, was present in SlDOF3.4 (Fig. S2B).

**Figure 1:**
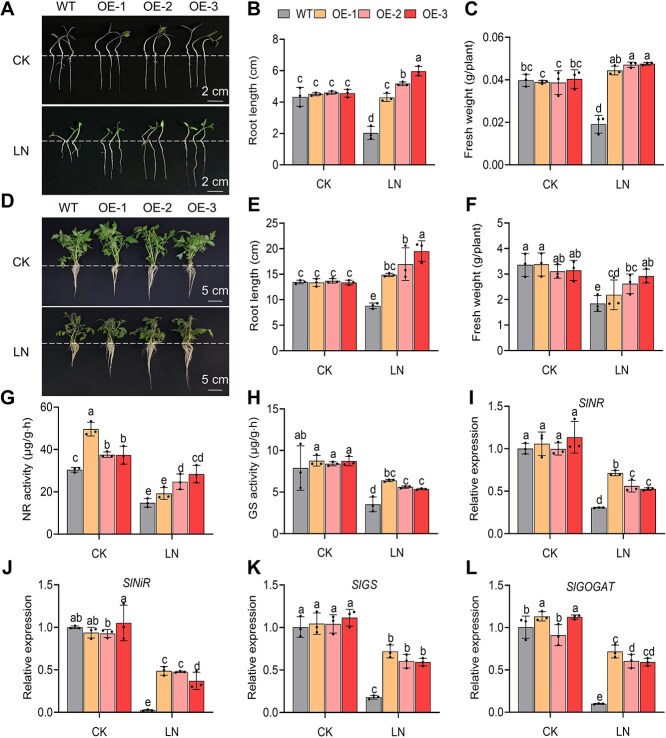
Overexpressing *SlDOF3.4* improves the growth of tomato seedlings under low N (LN) stress and upregulates the expression of genes related to N assimilation. (A–C) Representative photographs (A), root length (B), and fresh weight (C) of seedlings from wild-type (WT) and *SlDOF3.4*-OE lines OE-1, OE-2, and OE-3 after seed germination. Scale bars, 2 cm. (D–F) Representative photographs (D), root length (E), and fresh weight (F) of WT, *SlDOF3.4*-OE (OE-1, OE-2, and OE-3) seedlings grown under control (CK, 9 mM nitrate) or LN (0.2 mM nitrate) conditions for 7 days. (G–L) Nitrate reductase (NR) activity (G), glutamine synthetase (GS) activity (H), and relative expression levels of *SlNR* (I), *SlNiR* (J), *SlGS* (K), and *SlGOGAT* (L) in tomato seedlings grown under CK or LN conditions. Scale bars, 5 cm. Different lowercase letters indicate statistically significant differences (two-way analysis of variance [ANOVA] followed by Duncan's multiple range test, *P* < 0.05). Data are presented as means ± SD from three replicates.

To investigate the function of SlDOF3.4, we generated transgenic tomato lines overexpressing *SlDOF3.4* (*SlDOF3.4*-OE). We verified the transformants by genomic PCR and RT-qPCR (Fig. S3) and chose three homozygous lines (OE-1, OE-2, and OE-3) for analysis. We initially germinated seeds for these lines and the WT cultivar ‘Ailsa Craig’ under control nitrate conditions (9 mM nitrate, CK) before transferring the germinated seeds to filter paper soaked with CK or LN (0.2 mM nitrate) solution [29–31], followed by 7 days of growth. There were no significant differences in root length or fresh weight between WT and OE plants under CK conditions. However, root length and fresh weight were significantly greater in OE plants than in WT plants under LN conditions (Fig. 1A–C). In fact, the growth of young *SlDOF3.4-*OE seedlings under LN conditions was equivalent to or greater than that under CK conditions.

We obtained similar results when plants were grown over a longer period. Again, there were no significant differences in root length or fresh weight between 3-week-old WT and *SlDOF3.4*-OE plants under CK conditions, but both values were significantly higher in the OE lines than the WT under LN conditions (Fig. 1D–F). In addition, we also measured the nitrate nitrogen (${\mathrm{NO}}_3^{-}$-N) content in tomato seedlings under LN stress. As shown in Fig. S4, the overexpression of *SlDOF3.4* significantly increased the ${\mathrm{NO}}_3^{-}$-N content under LN conditions.

We examined how overexpressing *SlDOF3.4* promotes plant growth under LN conditions by measuring the activities of NR and GS and the expression levels of genes encoding key N-assimilation enzymes. Although NR and GS activities were lower under LN treatment than under control conditions, they were notably higher in the *SlDOF3.4-*OE lines than in the WT (Fig. 1G and H). Similarly, the expression levels of *SlNR*, *SlNiR*, *SlGS*, and *SlGOGAT* were lower in WT and *SlDOF3.4-*OE seedlings under LN conditions than under control conditions, with significantly higher expression levels in the *SlDOF3.4-*OE lines than the WT (Fig. 1I–L). These results strongly suggest that SlDOF3.4 enhances tomato growth under LN conditions by regulating the expression of genes related to N-assimilation.

### SlDOF3.4 binds to the promoters of *SlGS* and *SlLOXD*

**Figure 2:**
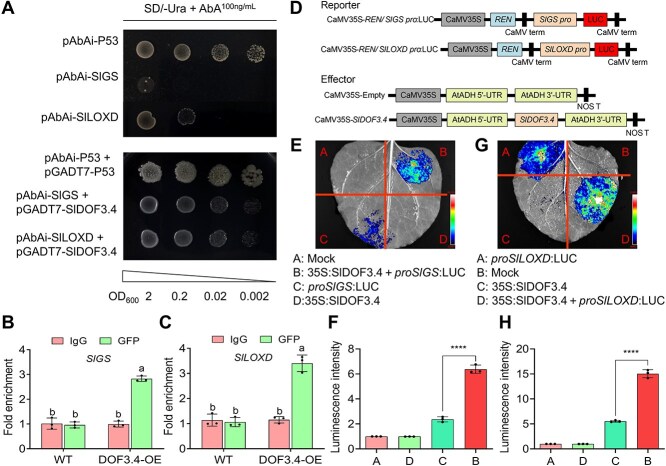
SlDOF3.4 directly binds to the promoters of *SlGS* and *SlLOXD* and activates their expression. (A) Yeast one-hybrid (Y1H) assay demonstrating that SlDOF3.4 binds to the promoters of *SlGS* and *SlLOXD*. SD, synthetic defined medium. (B, C) Chromatin immunoprecipitation quantitative PCR (ChIP-qPCR) assays showing the enrichment of SlDOF3.4 at the *SlGS* (B) and *SlLOXD* (C) promoters. (D) Diagrams of the reporter and effector constructs used for the dual luciferase assay. (E–H) Dual-luciferase reporter assays in *N. benthamiana* leaves showing that SlDOF3.4 activates the transcription of the promoters of *SlGS* and *SlLOXD.* Representative luminescence images (E, G) and relative firefly luciferase (LUC) activity upon co-infiltration of *35S:SlDOF3.4* with proS*lGS:LUC* (F) or *proSlLOXD:LUC* (H). Different lowercase letters indicate statistically significant differences (two-way ANOVA followed by Duncan's multiple range test, *P* < 0.05). Data are presented as means ± SD from three replicates.

DOFs recognize the *cis*-element 5′-(A/T)AAAG-3′ in the promoters of their target genes [13]. We noted that an AAAG motif was present in the promoters of the N-assimilation gene *SlGS* and the JA biosynthesis gene *SlLOXD*. To determine whether SlDOF3.4 directly regulates the expression of these genes, we examined its potential binding to these promoters. In yeast one-hybrid (Y1H) assays, yeast harboring the plasmid pAbAi-*SlGS* or pAbAi-*SlLOXD* and pGADT7-*SlDOF3.4* grew well on synthetic defined medium lacking uracil (SD/-Ura) containing 100 ng/ml aureobasidin A (AbA) (Fig. 2A). We verified these interactions by performing chromatin immunoprecipitation quantitative PCR (ChIP-qPCR) assays using WT seedlings and seedlings overexpressing *SlDOF3.4-GFP* (encoding a fusion of SlDOF3.4 and green fluorescent protein). SlDOF3.4 was enriched at the promoters of *SlGS* and *SlLOXD* (Fig. 2B and C). We established that SlDOF3.4 modulates the transcription of the *SlGS* and *SlLOXD* promoters using a dual-luciferase reporter assay in *Nicotiana benthamiana* leaves. We detected strong luminescence activity only when the *35S:SlDOF3.4* construct was co-infiltrated with the reporter construct *proSlGS:LUC* or *proSlLOXD:LUC* driving the expression of firefly luciferase (LUC) from the *SlGS* or *SlLOXD* promoter in *N. benthamiana* leaves (Fig. 2D–H). Together, these results indicate that SlDOF3.4 binds to the promoters of *SlGS* and *SlLOXD* and activates their transcription.

### JA content and JA-related gene expression in *SlDOF3.4-*OE plants

**Figure 3:**
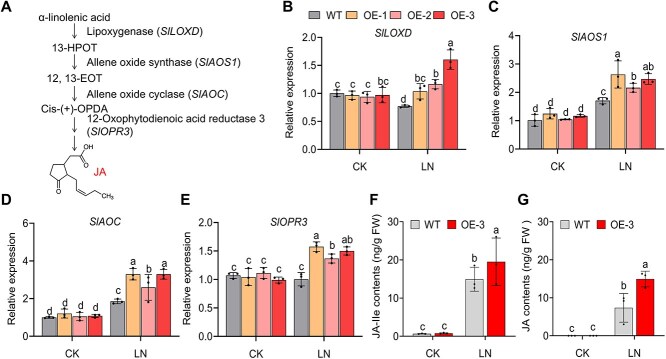
Overexpressing *SlDOF3.4* induces the expression of genes related to JA biosynthesis and increases the JA content of tomato seedlings under LN stress. (A) Diagram summarizing the enzymes and intermediates in the JA biosynthetic pathway. (B–E) Relative expression levels of the JA biosynthesis genes *SlLOXD* (B), *SlAOS1* (C), *SlAOC* (D), and *SlOPR3* (E) in WT and *SlDOF3.4*-OE (OE-1, OE-2, and OE-3) seedlings grown under CK or LN (0.2 mM nitrate) conditions, as measured by RT-qPCR. (F, G) JA-IIe (F) and JA (G) contents of WT and *SlDOF3.4-*OE-3 seedlings grown under CK or LN conditions. Different lowercase letters indicate statistically significant differences (two-way ANOVA followed by Duncan's multiple range test, *P* < 0.05). Data are presented as means ± SD from three replicates.

Treatment with 25 μM methyl jasmonate (MeJA) markedly alleviated the damage caused by LN stress, leading to greater fresh weight and longer roots in tomato (Fig. S5). We observed the upregulation of JA biosynthesis genes and a higher JA content in *SlDOF3.4*-OE tomato seedlings compared to the WT under LN conditions. To examine the molecular basis of this phenomenon, we measured the relative expression levels of *SlLOXD*, *SlAOS1*, *SlAOC*, and *SlOPR3*, which encode key enzymes of JA biosynthesis, by RT-qPCR (Fig. 3A). All genes were significantly upregulated in *SlDOF3.4*-OE seedlings compared to the WT under LN treatment (Fig. 3B–E). We then measured the endogenous JA contents of WT and *SlDOF3.4*-OE seedlings after 24 h of LN or CK treatment. The contents of JA and its conjugate to isoleucine (JA-IIe) were significantly higher in both genotypes upon LN treatment and were significantly higher in *SlDOF3.4*-OE seedlings than in WT plants under LN conditions (Fig. 3F and G). Considering that cytokinins are regarded as a pivotal factor in nitrogen response [32–34], we consequently measured the relative expression levels of genes associated with cytokinin biosynthesis in this study, namely *SlIPT2*, *SlARR5*, *SlLOG8*, and *SlCYP735A*. As illustrated in Fig. S6, LN treatment led to an increase in the expression levels of these genes, and the expression levels in *SlDOF3.4*-OE were notably higher than those in the WT. These results provide additional evidence for the upregulation of JA biosynthesis in tomato seedlings experiencing LN stress, indicating that JA can act synergistically with cytokinins to enhance the tolerance of tomato seedlings to LN stress.

### SlDOF3.4 interacts with SlCDF4

**Figure 4:**
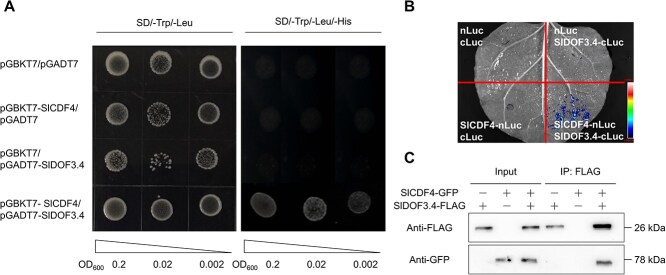
SlDOF3.4 physically interacts with SlCDF4. (A) Yeast two-hybrid (Y2H) assay demonstrating the interaction of SlDOF3.4 with SlCDF4. *SlDOF3.4* was cloned into the prey plasmid pGADT7, and *SlCDF4* was cloned into the bait plasmid pGBKT7. Yeast cells transformed with the indicated plasmid combinations were grown on synthetic define (SD) medium without leucine and tryptophan (SD/-Leu/-Trp) and without leucine, tryptophan, and histidine (SD/-Leu/-Trp/-His). pGADT7 and pGBKT7, empty vectors. (B) Luciferase complementation imaging (LCI) assay showing the interaction of SlDOF3.4 and SlCDF4 in plant cells. The full-length coding sequences of *SlDOF3.4* and *SlCDF4* were cloned individually in-frame and upstream of *cLUC* or *nLUC* to create the *SlDOF3.4-cLUC* and *SlCDF4-nLUC* constructs. The resulting vectors were co-infiltrated into the leaves of *N*. *benthamiana* plants. (C) Co-immunoprecipitation (Co-IP) assay showing the interaction of FLAG-tagged SlDOF3.4 with GFP-tagged SlCDF4. Proteins were detected using anti-GFP and anti-Flag antibodies.

DOF proteins interact with other TFs, including other members of the DOF family [11, 35, 36]. We therefore performed a yeast two-hybrid (Y2H) screen of a tomato leaf cDNA library to identify potential SlDOF3.4 interactors, one of which was the DOF protein SlCDF4. We confirmed the interaction between SlCDF4 and SlDOF3.4 by a directed Y2H assay, a luciferase complementation imaging (LCI) assay, and co-immunoprecipitation (Co-IP) assay. In the Y2H assay, yeast cells harboring pGADT7-*SlDOF3.4* and pGBKT7-*SlCDF4* grew well on SD/-Leu/-Trp/-His medium (Fig. 4A). In the LCI assay, the simultaneous expression of *SlDOF3.4-cLUC* and *SlCDF4-nLUC* produced strong LUC signals, indicating a direct interaction between SlCDF4 and SlDOF3.4 *in vivo* (Fig. 4B). We confirmed this *in vivo* interaction in a Co-IP assay using protein extracts from *N. benthamiana* leaves co-infiltrated with *35S:SlCDF4-GFP* and *35S:SlDOF3.4-FLAG* constructs; we detected SlCDF4-GFP among the proteins that co-precipitated with SlDOF3.4-FLAG following immunoprecipitation with an anti-FLAG antibody (Fig. 4C). These findings indicate that SlDOF3.4 physically interacts with SlCDF4 in cells and that SlDOF3.4-SlCDF4 might act together to regulate the expression of genes such as *SlGS* and *SlLOXD* during the LN response.

### The effects of *SlCDF4* overexpression are similar to those of *SlDOF3.4* overexpression

Phylogenetic analysis showed that SlCDF4 shared the highest identity with StCDF3 and was grouped into class I with AtCDF1, AtCDF2, and AtCDF3 from Arabidopsis (Fig. S2A). Multiple sequence alignment indicated that SlCDF4 contained the highly conserved zf-Dof domain (Fig. S7). We examined the expression of *SlCDF4* in the roots, stems, and leaves of tomato seedlings after exposure to 0.2, 2, or 9 mM nitrate for 24 h. *SlCDF4* expression was significantly higher (by 10% and 30%) in roots and 8.4-fold and 41.2-fold higher in leaves following exposure to 2 and 0.2 mM nitrate, respectively, compared to treatment with 9 mM nitrate (Fig. S8).

**Figure 5:**
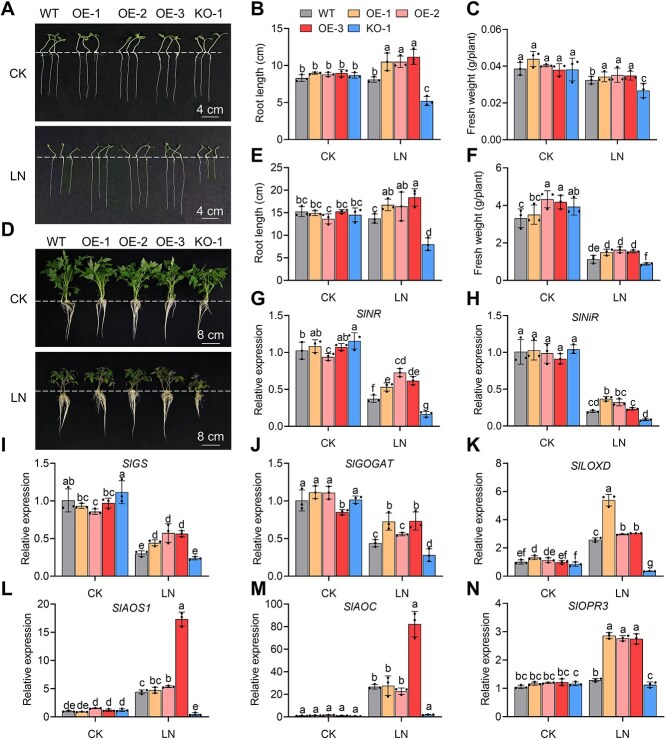
SlCDF4 improves tomato growth and promotes the expression of genes associated with N assimilation and JA biosynthesis under LN stress. (A–C) Representative photographs (A), root length (B), and fresh weight (C) of WT, *SlCDF4*-OE (OE-1, OE-2, OE-3), and *Slcdf4* KO-1 seedlings after seed germination. Scale bars, 4 cm. (D–F) Representative photographs (D), root length (E), and fresh weight (F) of 3-week-old WT, *SlCDF4*-OE, and *Slcdf4* KO-1 seedlings grown under CK or LN (0.2 mM nitrate) conditions for 7 days. Scale bars, 8 cm. (G–N) Relative expression levels of the N-assimilation genes *SlNR* (G), *SlNiR* (H), *SlGS* (I), and *SlGOGAT* (J) and the JA biosynthesis genes *SlLOXD* (K), *SlAOS1* (L), *SlAOC* (M), and *SlOPR3* (N) in WT, *SlCDF4*-OE, and *Slcdf4* KO-1 seedlings grown under CK and LN conditions for 7 days. Different lowercase letters indicate statistically significant differences (two-way ANOVA followed by Duncan's multiple range test, *P* < 0.05). Data are presented as means ± SD from three replicates.

To investigate the role of *SlCDF4* under LN conditions in more detail, we produced more than 20 *SlCDF4-*OE lines (Fig. S9) and obtained four independent *Slcdf4* knockout (KO) lines using clustered regularly interspaced short palindromic repeats (CRISPR)/CRISPR-associated nuclease 9 (Cas9)-mediated gene editing (Fig. S10). We chose *Slcdf4* KO-1, harboring a 5-bp deletion in the first target and a 1-bp insertion in the second target, for further analysis. We subjected seeds from WT, *SlCDF4-*OE, and *Slcdf4* KO-1 plants to CK or LN treatment for 7 days. Under CK conditions, there were no significant differences in root length or fresh weight among the genotypes. Under LN conditions, however, the *SlCDF4-*OE lines produced significantly longer roots and accumulated fresher biomass than WT seedlings, whereas *Slcdf4* KO-1 showed the opposite phenotype (Fig. 5A–C). Likewise, there were no significant phenotypic differences in root length among 3-week-old WT, *SlCDF4-*OE, and *Slcdf4* KO-1 seedlings under CK conditions. However, under LN conditions, the roots of *SlCDF4-*OE seedlings were longer, while those of *Slcdf4* KO-1 were shorter, compared to the WT, and fresh weight was lower in *Slcdf4* KO-1 seedlings compared to the other genotypes (Fig. 5D–F). The ${\mathrm{NO}}_3^{-}$-N content also demonstrated a similar trend, namely that the nitrate uptake capacity of *SlCDF4*-OE was significantly higher than that of WT and *Slcdf4*-KO (Fig. S11). Therefore, like *SlDOF3.4*, overexpressing *SlCDF4* improved plant growth in tomato under LN conditions.

We next examined whether SlCDF4 affects plant responses to LN stress by transcriptionally regulating key genes involved in N assimilation and JA biosynthesis. The expression levels of the N-assimilation genes *SlNR*, *SlNiR*, *SlGS*, and *SlGOGAT* and the JA biosynthesis genes *SlLOXD*, *SlAOS1*, *SlAOC*, and *SlOPR3* were indeed higher in the *SlCDF4-*OE lines than in the WT under LN conditions, whereas *Slcdf4* KO-1 seedlings showed the opposite patterns (Fig. 5G–N). The expression levels of genes related to cytokinin synthesis also exhibited a similar trend (Fig. S12). These results indicate that SlCDF4, like SlDOF3.4, promotes LN tolerance by regulating the expression of genes involved in N assimilation and JA biosynthesis.

### SlCDF4 also binds to the promoters of *SlGS* and *SlLOXD*

**Figure 6:**
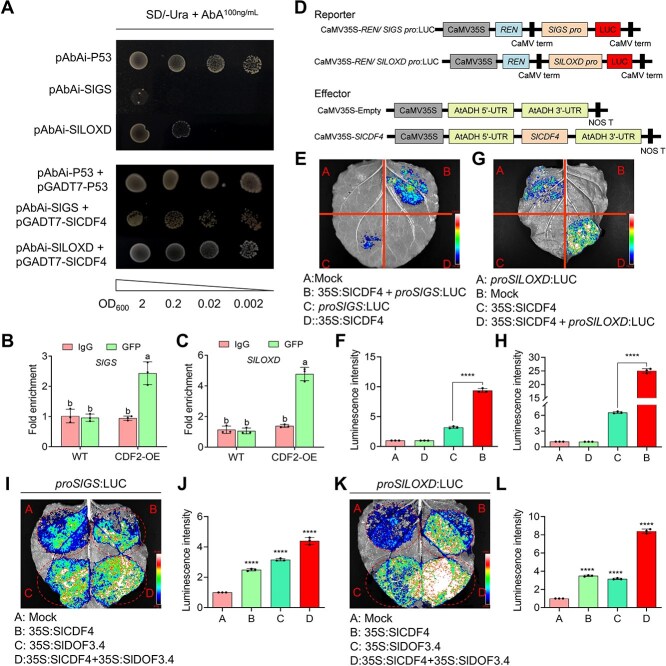
SlCDF4 directly binds to the promoters of *SlGS* and *SlLOXD*. (A) Y1H assay showing that SlCDF4 binds to the promoters of *SlLOXD* and *SlGS*. SD, synthetic defined medium. (B, C) ChIP-qPCR assay showing the enrichment of SlCDF4 at the *SlGS* and *SlLOXD* promoters. (D) Diagrams of the reporter and effector constructs used for the dual luciferase assay. (E–H) Dual-luciferase reporter assays in *N. benthamiana* leaves showing that SlCDF4 activates transcription from the promoters of *SlGS* and *SlLOXD*. Representative luminescence images (E, G) and relative luciferase activity upon co-infiltration of *35S:SlCDF4* with *proSlGS:LUC* (F) or *proSlLOXD:LUC* (H). (I–L) Dual-luciferase reporter assays showing the effect of co-infiltration of *35S:SlCDF4* and *35S:SlDOF3.4* on the transcriptional activation of the *SlGS* and *SlLOXD* promoters. Representative luminescence images (I, K) and relative luciferase activity upon co-infiltration of *35S:SlDOF3.4* and/or *35S:SlCDF4* with *proSlGS:LUC* (J) or *proSlLOXD:LUC* (L). Different lowercase letters indicate statistically significant differences (two-way ANOVA followed by Duncan's multiple range test, *P* < 0.05). Data are presented as means ± SD from three replicates.

As SlCDF4 interacts with SlDOF3.4 and plays similar roles to SlDOF3.4, we hypothesized that SlCDF4 would also activate *SlGS* and *SlLOXD* transcription. In Y1H assays, yeast cells grew well on SD/-Ura medium containing 100 ng/ml AbA when they carried the pAbAi-*SlGS* or pAbAi-*SlLOXD* plasmid together with pGADT7-*SlCDF4* (Fig. 6A). In ChIP-qPCR assays, SlCDF4 was enriched at the promoters of *SlGS* and *SlLOXD* (Fig. 6B and C). In LUC reporter assays with the effector construct *35S:SlCDF4* and the reporter constructs *proSlGS:LUC* and *proSlLOXD:LUC* [2000-bp upstream of the translation start site (Fig. 6D)], we detected strong LUC activity only when the *35S:CDF4* effector construct was co-infiltrated with the *proSlGS:LUC* or *proSlLOXD:LUC* reporter construct into *N. benthamiana* leaves (Fig. 6E–H). These results indicate that SlCDF4, like SlDOF3.4, binds to the promoters of *SlGS* and *SlLOXD* and activates their transcription.

We asked how the interaction between SlCDF4 and SlDOF3.4 might influence the transcriptional regulation of *SlGS* and *SlLOXD*. A LUC reporter assay revealed significantly enhanced LUC activity from the *proSlGS:LUC* and *proSlLOXD:LUC* reporters when *35S:SlCDF4* was co-infiltrated with *35S:SlDOF3.4* into *N. benthamiana* leaves (Fig. 6I–L).

### MeJA treatment reduces the sensitivity of *Slcdf4* KO plants to LN stress

**Figure 7:**
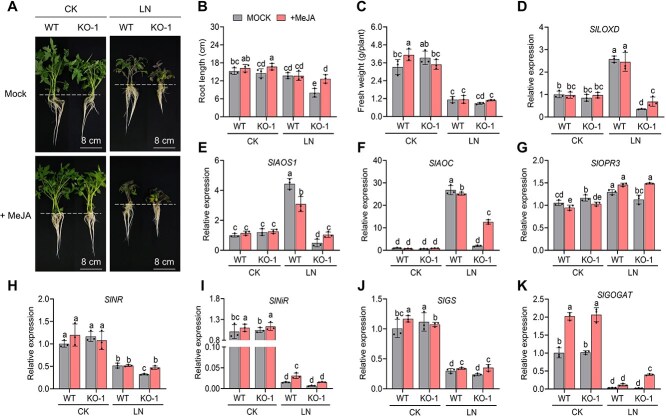
Effects of MeJA treatment on seedling growth and the expression of JA biosynthesis and N-assimilation genes in wild-type and *Slcdf4* KO-1 tomato seedlings under CK and LN conditions. (A–C) Representative photographs (A), root length (B), and fresh weight (C) of *Slcdf4* KO-1 and WT seedlings grown under CK or LN conditions following mock treatment or treatment with MeJA. Scale bars, 8 cm. (D–K) Relative expression levels of the JA biosynthesis genes *SlLOXD* (D), *SlAOS* (E), *SlAOC* (F), and *SlOPR3* (G) and the N-assimilation genes *SlNR* (H), *SlNiR* (I), *SlGS* (J), and *SlGOGAT* (K) in *Slcdf4* KO-1 and WT seedlings grown under CK or LN conditions following mock treatment or treatment with MeJA. Different lowercase letters indicate statistically significant differences (two-way ANOVA followed by Duncan's multiple range test, *P* < 0.05). Data are presented as means ± SD from three replicates.

To investigate the effect of JA on *Slcdf4* KO-1 plants under LN conditions, we sprayed 22.5 μM MeJA onto the leaves of WT and *Slcdf4* KO-1 tomato seedlings grown under CK or LN conditions. The WT and *Slcdf4* KO-1 seedlings looked essentially identical under CK conditions (Fig. 7A), while the *Slcdf4* KO-1 seedlings were more severely stunted than the WT under LN conditions. MeJA treatment alleviated some of the growth inhibition observed in *Slcdf4* KO-1 seedlings under LN stress. In particular, the roots of *Slcdf4* KO-1 seedlings were significantly longer than those of the mock treatment following MeJA treatment under LN conditions (Fig. 7B and C). In addition, the content of ${\mathrm{NO}}_3^{-}$-N in *Slcdf4* KO-1 seedlings treated with MeJA under LN stress was significantly higher than that in the control group (Fig. S13).

RT-qPCR analysis revealed that *SlLOXD*, *SlAOS1*, *SlAOC*, and *SlOPR3* were significantly downregulated in *Slcdf4* KO-1 compared to WT seedlings under LN conditions (Fig. 7D–G). However, MeJA treatment increased the expression levels of these genes in *Slcdf4* KO-1 compared to mock treatment. In addition, MeJA enhances cytokinins synthesis in tomato seedlings (Fig. S14). The expression levels of *SlNR*, *SlNiR*, *SlGS*, and *SlGOGAT* also increased following MeJA treatment in *Slcdf4* KO-1 plants (Fig. 7H–K). These results suggest that MeJA treatment reduces the sensitivity of *Slcdf4* KO-1 seedlings to LN stress by enhancing JA biosynthesis and promoting the expression of N-assimilation genes.

## Discussion

Understanding how plant genes respond to different N conditions is essential for formulating approaches, manipulating genes, and improving crop NUE. The cultivation of LN-tolerant crop varieties offers a potential solution to environmental pollution caused by the excessive use of N fertilizers [37]. There has been little research on the roles of DOF TFs in N metabolism in tomato, except for one study showing that heterologously expressing Arabidopsis *CDF3* in tomato resulted in greater biomass accumulation and enhanced root development under both N-poor and N-rich conditions [25]. In the present study, transgenic tomato seedlings overexpressing either *SlDOF3.4* or *SlCDF4* developed longer roots and produced higher fresh biomass under LN conditions than non-transformed WT seedlings, demonstrating a role for DOF proteins in N assimilation in tomato. These results are consistent with the previous findings that plant height, leaf surface area, total protein content, chlorophyll level, and sucrose and glucose contents were higher in *Dof1.7* transgenic tobacco (*Nicotiana tabacum*) plants than in WT plants [38]. We also determined that knocking out *SlCDF4* compromised the growth of tomato seedlings under LN conditions, providing evidence that *SlCDF4* improves growth in tomato. Unfortunately, we were unable to obtain knockout lines for *SlDOF3.4* for unknown reasons, possibly because this gene performs unknown essential functions.

In terms of the detailed mechanisms by which DOF proteins regulate N assimilation, previous studies have shown that CsDOF directly binds to the promoter of *CsGS2*, promoting its transcriptional activity [39] and CsDof16 directly binds to the −526 to −426 region of the *CsGS1.1* promoter, thereby activating its transcription and regulating N remobilization in tea (*Camellia sinensis*) plants [40], while PpDof5 from maritime pine (*Pinus pinaster*) activates the transcription of *GS1b* but transcriptionally represses *GS1a*^41^. Here, Y1H, LUC reporter, and ChIP-qPCR assays showed that both SlDOF3.4 and SlCDF4 directly bind to the *SlGS* promoter*,* activating its transcription and potentially promoting N assimilation. The conserved DOF domain mediates protein–protein interactions. In maize (*Zea mays*), DOF1 self-associates and interacts with the DOF protein DOF2 [11]. Interactions between DOFs and other types of TFs have also been reported, such as with basic leucine zipper (bZIPs) and MYB TFs^42^, as well as between Dof2.1 and ABRE-BINDING FACTOR 2 (ABF2) in sweet potato (*Ipomoea batatas*) [36] or DOF4.6 and NAC in Arabidopsis [35]. Our results provide another example of a DOF interactor *in vivo*: SlCDF4 interacts with SlCDF3.4, both of which bind to the *SlGS* promoter and activate its transcription. Such a regulatory module has not been reported previously and offers potential molecular targets for the improvement of growth in tomato. Our finding that co-expressing *SlCDF4* with *SlDOF3.4* enhanced the transcriptional activation of *SlGS* and *SlLOXD* provides support for our working model of the SlDOF3.4-SlCDF4 module.

Previous studies have revealed complex relationships among JA and DOF TFs: JA induces DOF gene expression in *Arabidopsis* [43]; TaDOF3A functions as a negative regulator and TaDOF5.6B as a positive regulator of JA biosynthesis in wheat [34]; OsDOF24 binds to the promoter of *OsAOS1* in rice [44]. CmDof13 was identified as a positive regulator of *CmLOX18* in melon fruit [45]. Little is known about the relationship between the LN response and activation of the JA pathway, although a recent study showed that N deficiency primes JA-mediated defense responses in eggplant (*Solanum melongena*), increasing herbivore resistance [46]. In the present study, the JA biosynthesis genes *SlLOXD*, *SlAOS1*, *SlAOC*, and *SlOPR3* were upregulated in *SlDOF3.4*-OE and *SlCDF4-*OE tomato seedlings under LN treatment, while *Slcdf4* KO plants showed the opposite effect. Because SlDOF3.4 and SlCDF4 bind to the *SlLOXD* promoter, as demonstrated in this study, we conclude that LN conditions likely induce JA biosynthesis in *SlDOF3.4*-OE and *SlCDF4-*OE tomato seedlings. Importantly, as MeJA treatment alleviated some of the sensitivity of *Slcdf4* KO seedlings to LN stress and upregulated genes associated with N assimilation, we further conclude that JA biosynthesis induced by LN treatment in *SlDOF3.4*-OE and *SlCDF4-*OE tomato seedlings represents a mechanism for the LN response. However, the molecular pathway by which JA signaling regulates N assimilation requires further study.

**Figure 8:**
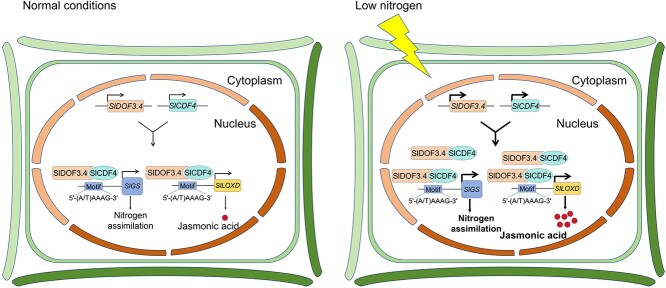
SlDOF3.4 interacts with SlCDF4 to promote LN tolerance by modulating the expression of genes related to N assimilation and JA biosynthesis in tomato. The expression of *SlDOF3.4* and *SlCDF4* increases under LN conditions, and SlDOF3.4 physically interacts with SlCDF4. SlDOF3.4 and SlCDF4 directly target and activate the N-assimilation gene *SlGS* and the JA biosynthesis gene *SlLOXD* and increase their expression, thereby enhancing LN tolerance.

In summary, we propose a working model based on the results of this study (Fig. 8). LN stress markedly induces the expression of both *SlDOF3.4* and *SlCDF4*. These two TFs, SlDOF3.4 and SlCDF4, are capable of specifically recognizing and binding to the 5′-(A/T)AAAG-3′ motifs present in the promoter regions of *SlGS* and *SlLOXD* genes, consequently regulating their transcriptional levels. Moreover, SlDOF3.4 and SlCDF4 can directly interact with one another. The interaction between them leads to the formation of the SlDOF3.4-SlCDF4 module, which demonstrates significantly enhanced transcriptional activation ability towards *SlGS* and *SlLOXD* compared to the individual factors. In terms of physiological functions, the SlDOF3.4-SlCDF4 module plays a dual role in enhancing the adaptability of tomato seedlings to LN stress. On the one hand, it boosts the nitrogen metabolism capacity of tomato seedlings under LN conditions, enabling more efficient utilization of the limited nitrogen resources. On the other hand, it promotes the biosynthesis of JA, thereby activating the JA-mediated LN response pathway. The combined effects of these two mechanisms substantially improve the tolerance of tomato seedlings to LN stress.

## Materials and methods

### Vector construction and plant transformation

The full-length coding sequences of *SlDOF3.4* (GenBank XM_004233894.5) and *SlCDF4* (GenBank XM_004233318.5) were individually amplified by PCR from cDNA of the tomato cultivar ‘Ailsa Craig’ and cloned into the pCAMBIA1301-YFP vector using a ClonExpress II One Step Cloning Kit (Vazyme, Nanjing, China). The *SlCDF4* knockout vector was constructed via CRISPR/Cas9-mediated genome editing [47]. The primers for the target genes were designed using the CRISPR-Cas9 primer design website (http://crispr.hzau.edu.cn/cgi-bin/CRISPR2/CRISPR). All primers used in this study are listed in Table S1. Recombinant plasmids were introduced into Agrobacterium (*Agrobacterium tumefaciens*) strain LBA4404 for transformation of tomato cultivar ‘Ailsa Craig’.

### Plant stress treatments

Tomato (*Solanum lycopersicum* L. cv. ‘Ailsa Craig’) seeds were surface sterilized by immersing in water at 55°C for 90 min. After surface sterilization, the seeds were rinsed thoroughly in running water and incubated at 28°C in the dark for 2 days. The germinated seeds were placed onto filter paper soaked with nutrient solution containing 9 mM nitrate (control; CK) or 0.2 mM nitrate (low nitrogen; LN) [29–31]; nitrate was provided as KNO_3_ and Ca(NO_3_)_2_. In a 1-l nutrient solution, the following chemical compounds are present: 590 mg of Ca(NO_3_)_2_·4H_2_O, 404 mg of KNO_3_, 136 mg of KH₂PO₄, 246 mg of MgSO₄·7H_2_O, 27.8 mg of FeSO₄, 37.2 mg of Na₂EDTA, 2.86 mg of H₃BO₃, 2.13 mg of MnSO₄·4H_2_O, 0.22 mg of ZnSO₄·7H_2_O, 0.08 mg of CuSO₄·5H_2_O, and 0.02 mg of (NH_4_)_6_Mo_7_O_24_·4H_2_O. Tomato seedlings were grown in a tissue culture room at a constant temperature of 24°C under a 16-h light/8-h dark photoperiod. After 7 days of treatment, the seedlings were photographed, and their root lengths, as well as the fresh weight of the whole plant, were recorded [47–49]. All experiments were set up with three independent biological replicates.

For the hydroponic culture experiments, 2-week-old tomato seedlings were grown hydroponically in plastic tanks containing 3 l of 0.2 mM nitrate solution (LN) or 9 mM nitrate solution (CK). After 7 days, root length and the fresh weight of the whole plant were recorded, and root and leaf samples were harvested and stored at −80°C for analysis [47]. All experiments consisted of three independent biological replicates.

MeJA was added to the CK and LN hydroponic solutions at concentrations of 10, 20, 25, 50, or 75 μM, and tomato seedlings were grown for 7 days as described above. The seedlings were photographed and their root lengths and the fresh weight of the whole plant were recorded. The leaves of WT and *slcdf4* KO plants grown under CK and LN conditions were sprayed with 22.5 μM MeJA. The seedlings were photographed, their root lengths and the fresh weight of the whole plant were measured, and the expression of genes related to N assimilation and JA biosynthesis was analyzed by RT-qPCR. All experiments consisted of three independent biological replicates.

### Quantitative reverse-transcription PCR (qPCR)

Total RNA was extracted from leaf, stem, and root samples from 2-week-old plants using a TransZol Up Plus RNA kit (TransGen, Beijing, China) following the manufacturer's instructions. First-strand cDNA was synthesized using a First-Strand cDNA Synthesis SuperMix for qPCR kit (TransGen, Beijing, China). Quantitative PCR (qPCR) was performed using Perfect Start Green qPCR SuperMix (Transgene, Beijing, China) on a BioRad CFX 96 Real-Time System. Relative expression levels were calculated using the 2^−ΔΔCt^ method. The primers used are listed in Table S2.

### NR and GS activity assays

NR and GS activities from the leaves of 2-week-old plants after control and LN treatment were measured as described by Xuekui and Huang [50].

### Yeast two-hybrid (Y2H) assay

A cDNA library was constructed from total RNA extracted from the leaves of 2-week-old tomato plants using the prey plasmid pGADT7. Yeast libraries were screened according to the manufacturer's instructions using the Matchmaker Gold Yeast Two-Hybrid System (Clontech, USA). The NCBI database was used to identify proteins that interacted with SlDOF3.4 based on the sequences of positive clones. To confirm the observed interaction between SlDOF3.4 and SlCDF4, the full-length coding sequence of *SlDOF3.4* was cloned into the pGADT7 plasmid, while the full-length coding sequence of SlCDF4 was cloned into the pGBKT7 plasmid, and both plasmids were introduced into yeast strain Y2HGold (Coolaber, Beijing) for growth on synthetic defined (SD) medium lacking tryptophan and leucine (SD/-Trp/-Leu) or SD/-Trp/-Leu/-His medium. All primers used are listed in Table S3.

### Luciferase complementation imaging assay

The full-length coding sequences of *SlCDF4* and *SlDOF3.4* were separately cloned into the pCAMBIA1301-cLUC and pCAMBIA1301-nLUC vectors to construct the cLUC-*SlCDF4* and nLUC-*SlDOF3.4* fusion expression vectors, respectively. The resulting plasmids were introduced into *Agrobacterium tumefaciens* strain GV3101. The bacterial cells were resuspended in buffer solution containing 10 mM magnesium chloride (MgCl_2_), 10 mM MES (2-(N-morpholino) ethanesulfonic acid), and 150 μM acetosyringone (AS), adjusting the optical density at 600 nm (OD600) to 0.8. After a 2-h incubation period, the bacterial suspension was infiltrated into the leaves of *Nicotiana benthamiana* plants. The LUC signals in the infiltrated regions were detected using a Tanon 5200 Multi Chemiluminescent Imaging System (Tanon, China) with D-luciferin Firefly (Gold Biotechnology, St. Louis, MO, USA). All primers used are listed in Table S4.

### Co-immunoprecipitation assay

The full-length coding sequences of *SlCDF4* and *SlDOF3.4* were inserted into the pRI101-GFP and pCAMBIA1301-FLAG vectors, respectively, and the resulting vectors were co-infiltrated into *N. benthamiana* leaves via Agrobacterium-mediated infiltration. The plants were incubated for 48 to 72 h. A Co-IP assay was then performed using a Beyotime Anti-Flag Affinity Gel Kit (Beyotime, Nanjing, China). All primers used are listed in Table S5.

### Measurement of JA contents

The contents of JA and JA-Ile were measured by Suzhou PANOMIX Biomedical Tech Co. (Suzhou, China). The leaves of 2-week-old tomato seedlings were frozen in liquid nitrogen and ground into a fine powder. Extracts from the samples were analyzed using a liquid chromatography-electrospray ionization-tandem mass spectrometry system (HPLC: Shimadzu Shim-pack UFLC CBM30A system; Shimadzu MS, Applied Biosystems 6500 Triple Quadrupole).

### Yeast one-hybrid (Y1H) assays

Promoter fragments (2000 bp upstream of the translation start site) of *SlGS* and *SlLOXD* were ligated into pAbAi, and the full-length coding sequences of *SlCDF4* and *SlDOF3.4* were ligated into pGADT7. The empty pGADT7 plasmid was used as a negative control. Appropriate pairs of plasmids were introduced into yeast strain Y1HGold (Coolaber, Beijing), and positive colonies were identified based on growth on SD/-Ura/-Leu medium. Positive colonies were resuspended, serially diluted, and spotted onto SD/-Ura/-Leu medium, with or without 100 ng/ml aureobasidin A (AbA). A Y1H assay was performed according to the manufacturer's instructions (Coolaber, China). A complete list of primers used can be found in Table S6.

### Luciferase reporter assay

The full-length coding sequences of *SlCDF4* and *SlDOF3.4* were cloned individually into pCAMBIA1301 to generate effector constructs driven by the CaMV 35S promoter. Promoter fragments (2000 bp) of *SlGS* and *SlLOXD* were inserted individually into the pRI101-LUC vector to serve as reporter constructs. The effector and reporter constructs were co-infiltrated into the leaves of 4-week-old *N. benthamiana* plants via Agrobacterium-mediated infiltration. Following treatment with D-luciferin (Gold Biotechnology, St. Louis, MO, USA), transcriptional activation was assessed by measuring firefly luciferase activity using a Tanon 5200 Multi Chemiluminescent Imaging System (Tanon, China). Primers used are listed in Table S7.

### ChIP-qPCR assays

ChIP-qPCR was performed using leaves from 4-week-old WT ‘Ailsa Craig’, *35S:SlDOF3.4-GFP*, and *35S:SlCDF4-GFP* tomato plants using a Simple ChIP Plus Sonication ChIP Kit (Cell Signaling Technology, USA). After recovering the precipitated complexes, ChIP-qPCR assays were performed using the primers listed in Table S8.

### Sequence alignment and phylogenetic analysis

In this study, multiple sequence alignment of the inferred protein sequences was conducted using ClustalX with the following parameters: a gap opening penalty of 5.0, a gap extension penalty of 0.2, and the delayed divergence of sequences with the negative matrix disabled [51]. A phylogenetic tree was constructed using the neighbor-joining method in MEGA 6 software, with 1000 bootstrap replicates performed and default parameters employed [52].

### Statistical analysis

All data were analyzed by one-way analysis of variance (ANOVA) using SPSS software (IBM, USA). The significance of differences between means was assessed using Duncan's multiple range test (*P* < 0.05). Error bars in all figures represent the standard deviation from the mean.

## Supplementary Material

Web_Material_uhag020

## Data Availability

All data used in this study are available in the article and its Supporting Information files.
