# Supplementary material for: The SlDOF3.4-SlCDF4 module improves tomato growth under low-nitrogen conditions
Source: Hortic Res. 2026 Jan 20;13(4):uhag020. doi: 10.1093/hr/uhag020 (PMC13108456; doi:10.1093/hr/uhag020)
Supplement: Web_Material_uhag020 [file web_material_uhag020.zip › 2025.12.18-SlDOF(supplemental materials).doc]

Table S1. The primers used for plasmid construction and transgenic tomato transformation.

| Vectors | Forward primer | Reverse primer |
| --- | --- | --- |
| pCAMBIA1301-YFP-*SlDOF3.4* (*Sma*I) | TACGAACGATACTCGACCCCATGACTTTGGAATCAAGTG | CTAGAGTCGACGGATCCCCCATTAAAATAATCTCCACTTC |
| pCAMBIA1301-YFP-*SlCDF4* (*Sma*I)  pHEE401E-*SlCDF4* (*Bsa*I-HF) | TACGAACGATACTCGACCCCATGACAGACCCCGCA  ATATATGGTCTCGATTGCAAAAAGTGAAGAAGAGCAGTTTTAGAGCTAGAAATAGC | CTAGAGTCGACGGATCCCCCTAAGCTCTCATTGAAAT  ATTATTGGTCTCGAAACCTCCTGGATCATCAATTCGCAATCTCTTAGTCGACTCTAC |

Table S2 RT-qPCR primer sequences.

| Gene | Forward primer | Reverse primer |
| --- | --- | --- |
| *SlActin* (Solyc11g005330.2.1) | CAGTTAAATCACGACCAGCAAGAT | TGTCCCTATTTACGAGGGTTATGC |
| *SlNR* (Solyc11g013810.2.1) | CCGGATCATGGTTTTCCGGT | TTGTACCACCAAGCTTCCGC |
| *SlNiR* (Solyc01g108630.3) | TTGGCGAGTGTGATAAGGAAA | GGCAGCACTACTCCACGAAT |
| *SlGS* (Solyc01g080280.3) | GGCGTGACACTGAGAAGGAA | GAGCTTCAGCCTCAAGGTT |
| *SlGOGAT* (Solyc03g063560.3.1) | GGTCTCGGGAGAACTAGGGT | CCCGAAGCTTGGCTCCATAA |
| *SlOPR3* (Solyc07g007870.3) | TCCGACTTCAGCTGGGTTTC | GAGATGCACGACCAACATGC |
| *SlLOXD* (Solyc03g122340.3) | CTGATGGTGTCATCGAGGCT | ACTGCCATCCCTCTTCGGAT |
| *SlAOS1* (Solyc04g079730.1) | GGGTCGGAAACTGAAAACGC | GAGGAACAGTTCGACCAGCA |
| *SlAOC* ([Solyc02g085730.3.1](http://plants.ensembl.org/Solanum_lycopersicum/Transcript/Summary?db=core;t=Solyc02g085730.3.1;tl=0u4Y8qskyTMd5fCV-24952033-2896490616)) | TGTCCCCTTTAGCAACAAGCTAT | ATCGCCGAAGTAGAAGCTGT |
| *SlIPT2*  ([Solyc04g007240.1.1](http://plants.ensembl.org/Solanum_lycopersicum/Transcript/Summary?db=core;t=Solyc04g007240.1.1;tl=wQlS4yfNCACjfDL1-24952062-2896493834)) | CTCGATTCGGAGATGTTTGA | TCTCGAACTCCGGTACTCCT |
| *SlCYP735A*  ([Solyc02g094860.2.1](http://plants.ensembl.org/Solanum_lycopersicum/Transcript/Summary?db=core;t=Solyc02g094860.2.1;tl=kc1q6D8q8UOmgSFQ-24952065-2896493852)) | GACAGAGCCGAGAATGTGT | AAGCAGGAGCAACAATGTG |
| *SlLOG8*  (Solyc08g062820.3.1) | GGTCTATGGTGGTGGAAGTG | GCAATAAAGGCATCAGCC |
| *SlARR5*  (Solyc05g006420.3.1) | ATAGTCATTGAGCGTCTCCTC | CAGTCATTCCAGGCATACAA |
| *SlDOF3.4*  (Solyc10g009360.3.1) | CAATTTGTCTCAGCCACGCC | GCGTGAGAAGAAACGGAAGC |
| *SlCDF4*  (Solyc02g067230.3.1) | TCTGGAGCTCAGGGAGATGA | AGGTTTCCTCCAAAGTCATCCTTA |

Table S3. Primers for Y2H.

| Vectors | Forward primer | Reverse primer |
| --- | --- | --- |
| pGADT7-*SlCDF4* (*Nde*I, *BamH*I) | GATTACGCTCATATGACAGACCCCGCAAT | GAGCTCGATGGATCCTAAGCTCTCATTGAAA |
| pGBKT7*-SlDOF3.4* (*Nde*I, *BamH*I) | GATTACGCTCATATGACTTTGGAATCAAGTG | GAGCTCGATGGATCCATTAAAATAATCTCCACTTC |

Table S4. Primers for LCI.

| Vectors | Forward primer | Reverse primer |
| --- | --- | --- |
| pCAMBIA1301-35S-cLUC- *SlDOF3.4* (*Kpn*I, *Sal*I) | TCCCGGGGCGGTACCATGACTTTGGAATCAAGTG | GCTCTGCAGGTCGACTTAATTAAAATAATCTCCACTTC |
| pCAMBIA1301-35S-*SlCDF4*-nLUC (*Kpn*I, *Sal*I) | GACGAGCTCGGTACCATGACAGACCCCGCAAT | CGAGATCTGGTCGACTAAGCTCTCATTGAAA |

Table S5. Primers for Co-IP.

| Vectors | Forward primer | Reverse primer |
| --- | --- | --- |
| *SlDOF3.4*-FLAG (*Nde*I, *Eco*RI) | CTGTTGATACATATGACTTTGGAATCAAGTG | ATCATCGATGAATTCATTAAAATAATCTCCACTT |
| *SlCDF4*-GFP (*Sma*I) | TACGAACGATACTCGACCCCATGACAGACCCCGCA | CTAGAGTCGACGGATCCCCCTAAGCTCTCATTGAAAT |

Table S6. Primers for Y1H.

| Vectors | Forward primer | Reverse primer |
| --- | --- | --- |
| pGADT7-*SlCDF4* (*Nde*I, *BamH*I) | GATTACGCTCATATGACAGACCCCGCAAT | GAGCTCGATGGATCCTAAGCTCTCATTGAAA |
| *SlLOXD pro*- pAbAi (*Hind*III, *Kpn*I) | TGAATTGAAAAGCTT  CTCACTTATTCTCTCCTCCAT | AGATCCCCGGGTACCCAAGGTGACAAATCAAATAAA |
| *SlGS pro*- pAbAi (*Hind*III, *Kpn*I)  pGADT7-*SlDOF3.4*(*Nde*I,*BamH*I) | TGAATTGAAAAGCTT CAAATAATGACAAAAGCC  GATTACGCTCATATGACTTTGGAATCAAGTG | AGATCCCCGGGTACCTTCTATATTTCTCCTATCATT  GAGCTCGATGGATCCATTAAAATAATCTCCACTTC |

Table S7. The primers used for dual luciferase reporter assay.

| Vectors | Forward primer | Reverse primer |
| --- | --- | --- |
| pCAMBIA1300-GFP-*SlCDF4* (*Sma*I) | TACGAACGATACTCGACCCCATGGAAGATGCTGCCTTTACG | CTAGAGTCGACGGATCCCCCGGCTGGACCGCTGCTACC |
| pCAMBIA1300-GFP-*SlDOF3.4* (*Sma*I) | TACGAACGATACTCGACCCC ATGGATGAACGCGTTATT | CTAGAGTCGACGGATCCCCCCTGTACTAATATAGGACTGCTGC |
| pRI101-*SlGS pro*-LUC (*Hin*dIII, *Sal*I) | TGAATTGAAAAGCTT CAAATAATGACAAAAGCC | AGATCCCCGGGTACCTTCTATATTTCTCCTATCATT |
| pRI101-*SlLOXD pro*-LUC (*Hin*dIII, *Sal*I) | GCCAGTGCCAAGCTTCTCACTTATTCTCTCCTCCAT | GTCTTCCATGTCGACCAAGGTGACAAATCAAATAAA |

Table S8. Primers for ChIP-qPCR analysis.

| Gene | Forward primer | Reverse primer |
| --- | --- | --- |
| *SlLOXD* | GTTATCGTGTTATCGGTTCTT | CTCTAAGTTGAGGACTTTATATACA |
| *SlGS* | GAATCTGGTAAAGGGTGACCTTAT | GAAGAAGATGAAGATGGGTGAGTA |

Figure S1. Expression of *SlDOF3.4* in leaf **(A)**, stem **(B)**, and root **(C)** of tomato seedlings exposed to 0.2 mM, 2 mM, or 9 mM (CK) nitrate for 24 h, as measured by RT-qPCR. Error bars represent the standard deviations (SDs) of the measurements from three independent experiments. The different lowercase letters indicate significant difference at *P <* 0.05 based on a one-way ANOVA test.

Figure S2. Bioinformatics analysis of SlDOF3.4. **(A)** Phylogenetic relationships between Dof family members in tomato, potato and Arabidopsis. SlDOF3.4 was marked with a star. **(B)** Multiple sequence alignment of the conserved Dof domain in 12 Dof proteins. The zf-Dof domain are shown. Identical amino acids are highlighted.

Figure S3. The genomic PCR and RT-qPCR analysis of *SlDOF3.4* overexpression (OE) plants. **(A)** The genomic PCR analysis. P: pCAMBIA-*SlDOF3.4* plasmid. WT: wild type. 1-10 means different OE lines. Marker: DNA Marker. **(B)** RT-qPCR analysis of OE plants. Error bars represent the standard deviations (SDs) of the measurements from three independent experiments. The different lowercase letters indicate significant difference at *P <* 0.05 based on a one-way ANOVA test.

Figure S4. The nitrate nitrogen content in tomato seedlings under low-nitrogen stress.

Error bars represent the standard deviations (SDs) of the measurements from three independent experiments. The different lowercase letters indicate significant difference at *P* < 0.05 based on a two-way ANOVA test.

Figure S5. Effect of exogenous MeJA on tomato growth under low nitrogen stress. MeJA of 10, 20, 25, 50, 75µM was added to 0.2 mM low nitrogen solution. Phenotype **(A)**, plant height **(B)**, Dry weight **(C)**, fresh weight **(D)** and root length **(E)** of tomato after different MeJA treatment under 0.2 mM low nitrogen. Error bars represent the standard deviations (SDs) of the measurements from three independent experiments. The different lowercase letters indicate significant difference at *P <* 0.05 based on a one-way ANOVA test.

Figure S6. The relative expression levels of genes involved in the synthesis of cytokinins substances under low-nitrogen stress. The relative expression levels of *SlIPT2* **(A)**, *SlARR5* **(B)**, *SlLOG8* **(C)**, *SlCYP735A* **(D)**. Error bars represent the standard deviations (SDs) of the measurements from three independent experiments. The different lowercase letters indicate significant difference at *P* < 0.05 based on a two-way ANOVA test.

Figure S7. Multiple sequence alignment of SlCDF4 of the conserved Dof domain. The zf-Dof domain are shown. Identical amino acids are highlighted.

Figure S8. Expression of *SlCDF4* in root **(A)**, stem **(B)**, and leaf **(C)** of tomato seedlings exposed to 0.2 mM, 2 mM, or 9 mM (CK) nitrate for 24 h, as measured by RT-qPCR. Error bars represent the standard deviations (SDs) of the measurements from three independent experiments. The different lowercase letters indicate significant difference at *P <* 0.05 based on a one-way ANOVA test.

Figure S9. The genomic PCR and RT-qPCR analysis of *SlCDF4* overexpression (OE) plants. **(A)** The genomic PCR analysis. P: pCAMBIA-*SlCDF4* plasmid. WT: wild type. **(B)** RT-qPCR analysis of OE plants. Error bars represent the standard deviations (SDs) of the measurements from three independent experiments. The different lowercase letters indicate significant difference at *P <* 0.05 based on a one-way ANOVA test.

Figure S10. The characterization of *slcdf4* knockout transgenic tomato by sequence analysis.

Figure S11. The nitrate nitrogen content in tomato seedlings under low-nitrogen stress. Error bars represent the standard deviations (SDs) of the measurements from three independent experiments. The different lowercase letters indicate significant difference at *P* < 0.05 based on a two-way ANOVA test.

Figure S12. The relative expression levels of genes involved in the synthesis of cytokinins substances under low-nitrogen stress. The relative expression levels of *SlIPT2* **(A)**, *SlARR5* **(B)**, *SlLOG8* **(C)**, *SlCYP735A* **(D)**. Error bars represent the standard deviations (SDs) of the measurements from three independent experiments. The different lowercase letters indicate significant difference at *P* < 0.05 based on a two-way ANOVA test.

Figure S13. The nitrate nitrogen content in tomato seedlings under low-nitrogen stress with MeJA treatment. Error bars represent the standard deviations (SDs) of the measurements from three independent experiments. The different lowercase letters indicate significant difference at *P* < 0.05 based on a two-way ANOVA test.

Figure S14. MeJA enhances cytokinins synthesis in tomato seedlings under low nitrogen conditions. The relative expression levels of *SlIPT2* **(A)**, *SlARR5* **(B)**, *SlLOG8* **(C)**, *SlCYP735A* **(D)**. Error bars represent the standard deviations (SDs) of the measurements from three independent experiments. The different lowercase letters indicate significant difference at *P* < 0.05 based on a two-way ANOVA test.
